# Malapposition of graft-host interface after penetrating keratoplasty (PK) and deep anterior lamellar keratoplasty (DALK): an optical coherence tomography study

**DOI:** 10.1186/s12886-020-1307-7

**Published:** 2020-01-31

**Authors:** Yujin Zhao, Hong Zhuang, Jiaxu Hong, Lijia Tian, Jianjiang Xu

**Affiliations:** 1 Department of Ophthalmology and Visual Science, Eye, and ENT Hospital, Shanghai Medical College, Fudan University, 83 Fenyang Road, Shanghai, China; 2 Key NHC Key Laboratory of Myopia (Fudan University); Laboratory of Myopia, Chinese Academy of Medical Sciences, Shanghai, China; 3 Key Laboratory of Myopia, National Health and Family Planning Commission, Shanghai, China; 4 Leiden Academic Centre for Drug Research, Leiden, The Netherlands

**Keywords:** Keratoconus, Penetrating keratoplasty, Deep anterior lamellar keratoplasty, Graft-host interface, Anterior segment optical coherence tomography, Visual outcome

## Abstract

**Backgroud:**

Previous studies of internal graft-host malappositions have not dealt with the precise ways in which each malapposition affected post-penetrating keratoplasty (post-PK) visual outcomes. In this study, we reviewed our post-PK and post-deep anterior lamellar keratoplasty (post-DALK) keratoconic patients and used anterior segment optical coherence tomography (AS-OCT) to evaluate the associations between graft-host interface (GHI) characteristics and visual outcomes.

**Methods:**

Novel GHI metrics included: mean graft-host touch (GHT), total prevalence of malapposition proportion (Pm), frequency of apposition (F), size of malapposition (Sm), junctional graft thickness (Tg), junctional host thickness (Th) and the absolute value of difference between Tg and Th (|Tg-Th|). We connected the external and internal junction points of GHI (GHT) and drew a straight line through the central point, perpendicular to both sides of the cornea. Tg and Th were the thicknesses at cross-points 1 mm away from the meeting point on the external side of the graft and host, respectively. Linear regression analysis was used to describe associations between GHI metrics and postsurgical visual outcomes [logarithm of minimum angle of resolution best-corrected visual acuity (logMAR BCVA), spherical equivalent diopter (SE), diopter of spherical power (DS), diopter of cylindrical power (DC) and keratometric astigmatism (Astig value)].

**Results:**

We enrolled 22 post-PK and 23 post-DALK keratoconic patients. Compared with the regular-apposition results, GHT was decreased in step and gape patterns, and increased in hill and tag patterns. SE increased averagely by 6.851, 5.428 and 5.164 diopter per 1% increase in: F (step) [β = 6.851; 95% Confidence interval (CI) = 2.975–10.727; *P* = 0.001]; F (graft step) [β = 5.428; 95% CI = 1.685–9.171; *P* = 0.005]; and Pm [β = 5.164; 95%CI = 0.913–9.146; *P* = 0.018], respectively. SE increased averagely by 0.31 diopter per 10-μm increment in |Tg-Th| [β = 0.031; 95% CI = 0.009–0.054; *P* = 0.007]. LogMAR BCVA increased (on average) by 0.01 per 10-μm increment in both GHT [β = 0.001; 95% CI = 0–0.002; *P* = 0.030]. and Tg [β = 0.001; 95% CI = 0.001–0.002; *P* = 0.001]. Astig value increased on average by 0.17 diopter per 10-μm increment in Sm [β = 0.017; 95% CI = 0–0.033; *P* = 0.047].

**Conclusion:**

This investigation of GHI characteristics suggests explanations for varied ametropia in keratoconic eyes and has potential significance as a reference for promoting pre-surgical planning and technology for corneal transplantation.

## Background

Patients with advanced keratoconus usually have very low vision because to progressive high myopia and irregular astigmatism. Therefore, the primary objective of keratoplasties for these patients should be rebuilding normal corneal curvature to provide acceptable vision [1, 2]. Penetrating keratoplasty (PK) and deep anterior lamellar keratoplasty (DALK) are two main surgical treatments for keratoconus. Despite progress in surgical and examination techniques, postoperative ametropia, and the attendant suboptimal vision, continues to occur; the reasons for this are unclear [3]. Approximately 40% of patients experienced astigmatism after keratoplasty. Among those who had astigmatism, 19 to 38% suffered from high astigmatism (> 5 diopters), which could not be satisfactorily corrected with spectacles or contact lenses [4–7]. Because keratoconus usually affects young and middle-aged people, achieving satisfactory vision for a lifetime should be the ultimate goal of surgical treatment [2, 3, 5, 6, 8].

Anterior-segment optical coherence tomography (AS-OCT) can penetrate through deep tissues with a stretched wavelength (1.3 μm). AS-OCT can safely be applied at the perioperative stage to obtain cross-sectional images of the anterior segments, which is critical for preoperative evaluation and postoperative follow-ups [9]. Therefore, AS-OCT has been used to observe alignment patterns of the posterior graft-host junctions after PK. However, similar observations after DALK have rarely been reported [10, 11].

To-date, knowledge of the factors influencing postsurgical refractive errors has remained inconclusive because of insufficient relevant reports. Kaiserman and Bahar [12] reported that internal graft-host malappositions were associated with increased postoperative ametropia. However, the precise way in which each malapposition affected postsurgical visual outcomes has rarely been reported [13]. Thus, the aim of this study was to analyze the associations between characteristics of the posterior graft-host interfaces (GHI) and the postoperative visual outcomes in keratoconus patients.

## Methods

### Patients

A retrospective observational cross-sectional study was conducted from March to August 2016 at the Eye & ENT Hospital of Fudan University, Shanghai, China. All analyses were performed retrospectively, based on review of medical records of patients who had surgical treatments (PK or DALK). All subjects received comprehensive ophthalmologic examinations including visual acuity, intraocular pressure, anterior segment photographs, slit-lamp biomicroscopy, and refraction. Visual acuity was measured with Snellen charts. Only patients without any postsurgical complications such as secondary glaucoma, cataract, or iris synechia were included. According to the widely known Amsler-Krumeich classification, subjects with grade 4 keratoconus, but no histories of ophthalmic diseases, surgeries, or trauma, were enrolled [14]. Written informed consent was obtained from every subject for the participation in the study. This investigation adhered to the tenets of the Declaration of Helsinki and was approved by the Ethics Committee of Shanghai Eye & ENT Hospital of Fudan University.

### Surgical technique

All corneal transplantations were performed by skilled ocular anterior segment surgeons (X.J.J., Z.Z.R. et al.) from our hospital who each have more than 20 years’ corneal surgery experience. Fresh full-thickness cornea materials that preserved and provided by the local eye bank were used. All surgeries were performed after complete akinesia of the eyeballs together with eyelids under retrobulbar and peribulbar anaesthesia. PKs were performed using standard techniques. The diameter of each recipient bed (7.50 mm to 8.25 mm) was decided according to preoperative AS-OCT examinations. The donor was excised to the same diameter as the recipient using a manual trephine system. Intraocular viscoelastic injections (VISCOAT®; Alcon Laboratories Ltd., Ft. Worth, TX) were applied to protect corneal endothelium of grafts. The graft was secured to the host bed using 12 or 16 interrupted 10–0 nylon non-absorbable surgical suture (USIOL, Inc.; Lexington, KY), according to the surgeon’s preference. At the end of each operation, anterior chamber was restored by saline injection and the watertightness of corneal wound was carefully checked. In DALK procedure, the diameter range (7.50 mm to 8.25 mm) of host bed was similar to that in PK. After a partial trephination of approximately 50% of the host corneal stroma and manual stripping of the superficial stromal layer, pneumatic pressure was used to detach the Descemet’s membrane (DM) from the deep stroma by injecting sterile air into the latent space between these two adjacent corneal microstructures with a 30-gauge needle. Air injection would produce a dome-shaped bubble that could be seen under the surgical microscope. Corneal stromal tissue above the “bubble” was manually dissected with scissors and spatula until a complete exposure of DM underneath was achieved. The same-size donor without DM and endothelium was then sutured to the recipient using a 12- or 16-bite interrupted suturing technique.

## Instruments and methods

All scans were performed in dark environment. Subjects were required to look straight at a fixation target inside of the device with their eyes opened widely by a blepharostat and to sit motionless throughout the examination. Only when subject’s eyeball position was stable, did we perform scanning.

### Oculus analysis

Corneal topography images were automatically taken by the system software (OCULUS Keratograph® 5 M; OCULUS, Wetzlar, Germany) when the best manual adjustments of eyeball position were acquired by one single ophthalmologist (Z.Y.J). Data from images in which cornea coverage was greater than 70% was used in the following statistical procedures. Each parameter was an average of three consecutive measured values and was collected by one single examiner (W.D)

### As-OCT imaging

Visante™ AS-OCT (Carl Zeiss Meditec, Inc.; Dublin, CA), a noninvasive non-contact optical tomography technology, was used in our study. Each corneal graft underwent four high-resolution optical sections that separated by 45 degrees, so that eight GHI sections were obtained (Additional file 1: Figure S1). One of the scan axes was placed on the main corneal meridian according to the previously determined directions on the OCULUS.

For consistency, OCT images were acquired and interpreted by a single ophthalmologist (Z.Y.J). Based on definitions from a previous report [12], graft-host alignment patterns were classified according to the internal surfaces of the corneal wound as follows: regular-apposed junctions (Additional file 2: Figure S2A), step [graft step (Additional file 2: Figure S2B) and host step (Additional file 2: Figure S2C)], protrusion [hill (Additional file 2: Figure S2D) and tag (Additional file 2: Figure S2E)], and gape (Additional file 2: Figure S2F).

### Statistical parameters

The post-surgery visual outcomes were assessed by five refraction parameters: logarithm of minimum angle of resolution best-corrected visual acuity (logMAR BCVA), spherical equivalent diopter (SE), diopter of spherical power (DS), diopter of cylindrical power (DC) and Astig value (keratometric astigmatism). Astig value was an inherent keratometric parameter in OCULUS system and represented the keratometric astigmatism in the central 2 mm of the cornea. To describe the characteristics of the postoperative GHI, novel AS-OCT parameters were defined as below: mean graft-host touch (GHT), total prevalence of malapposition proportion (Pm), frequency of apposition (F), size of malapposition (Sm), junctional graft thickness (Tg), junctional host thickness (Th), and the absolute value of the difference between Tg and Th (|Tg-Th|). To clarify, GHT, Sm, Tg, Th, and |Tg-Th| were all arithmetic means derived from date from eight scan points in each cornea (Table 1; Additional file 3: Figure S3 and Additional file 4: Figure S4).

**Table 1:** Definitions and Measurement Methods of AS-OCT Parameters

Parameters	Definition	Measurement methods
Pm	Total prevalence of malapposition proportion: proportion of all malappositions in one eye (range:1/8~8/8)	Divide the number of malapposition in one eye by 8, then multiplied by 100%
F	Frequency of apposition: proportion of a certain type of apposition in one eye (range: 1/8~8/8)	Divide the total number of a type of apposition in one eye by 8, then multiplied by 100%
GHT	Mean graft-host touch: the linear distance between epithelial and endothelial surfaces of the GHI	Linear distance between the external and internal junction points of GHI
Sm	Size of malapposition: the maximum distance between malapposition and the normal DM layer	1.Gape of protrusion (hill and tag) alignment: through the vertex of protrusion or gape, draw a straight line that is perpendicular to the DM layer or tangential direction of DM layer, then record the length
		2.Graft step alignment: first locate the farthest point on graft step from the host, then make a virtual extension curve from the host DM layer, and then draw a straight line that is perpendicular to the tangential direction of the curve and record the length. Host step alignment: refer to the procedure for graft step alignment.
Tg and Th	Junctional graft thickness (Tg): graft thickness 1 mm from GHI; Junctional host thickness (Th): host thickness 1 mm from GHI	First connect the external and internal junction points of GHI and locate a central point, then draw a straight line through the point and make it perpendicular to both external and internal sides of the cornea. Calculate Tg and Th by measuring the thicknesses at cross-points 1 mm away from the meeting point on the external side of graft and host sides.
|Tg-Th|	absolute value of difference between Tg and Th	mathematical calculation

*DM* Descemet’s membrane, *GHI* Graft-host interface

### Statistical analysis

Data were analyzed using SPSS® version 19 (IBM® Corp.; Armonk, NY). All measurements were expressed as mean ± standard deviation (SD). After estimating the normality and homoscedasticity of all the data using the Shapiro-Wilk normality test and Levene test, one-way analysis of variance was used for continuous variables, while ranked data were analyzed by Mann-Whitney U test or Kruskal-Wallis test. Pearson and the Spearman correlations were employed to assess the relationships between post-surgical visual outcomes and GHI characteristics. Linear regression analysis was performed to establish equations for visual outcome parameters which showed significant correlations with AS-OCT parameters. Probabilities of less than 5% were considered statistically significant.

## Results

A total of 360 graft-host sections from 45 AS-OCT images of 45 eyes (40 male and 5 female) were acquired and analyzed, including 22 post-penetrating keratoplasty (post-PK) eyes and 23 post-deep anterior lamellar keratoplasy (post-DALK) eyes. Demographics, postoperative visual outcomes and part of AS-OCT parameters were compared between post-PK and post-DALK (Table 2). The mean follow-up time for the DALK group was significantly shorter than that for the PK group (20.82 ± 10.54 vs. 38.18 ± 22.91, respectively, *P* = 0.003). DALK patients had relatively higher (worse) logMAR BCVA and lower SE and DS values than did the PK patients. Tg was greater in the post-DALK group than in the post-PK group. Correspondingly, the |Tg-Th| value in the DALK group was larger. Among other GHI metrics, GHT, Pm, and Sm showed no significant differences between groups. However, the frequency of different types of apposition differed between the groups. Post-DALK eyes had more step and graft-step alignments, whereas post-PK eyes had more host, hill and tag junctions. Hill alignment was not observed in post-DALK eyes. The frequencies of regular-apposition and gape did not differ significantly between groups (Table 2 and Table 3; Additional file 5: Figure S5).

**Table 2:** Comparisons of Demographics and Postoperative Parameters between Post-PK and Post-DALK groups

	Post-PK	Post-DALK	P
	Mean (SD)	Mean (SD)	
Age (Years)	25.82 (7.29)	22.35 (6.00)	.088
Mean follow-up time (Months)	38.18 (22.91)	20.82 (10.54)	.003
IOP (mmHg)	18.13 (1.29)	17.74 (1.68)	.838
logMAR BCVA	0.25 (0.24)	0.47 (0.20)	.002
SE (diopter)	−4.59 (4.74)	−1.29 (3.75)	.013
DS (diopter)	−2.35(4.55)	0.81 (3.29)	.010
DC (diopter)	−4.47(2.64)	−4.19 (2.57)	.720
Astig value (diopter)	0.05 (4.35)	0.72 (4.53)	.618
GHT (μm)	686.50 (102.09)	679.52 (52.84)	.778
Pm (%)	54.5 (30.5)	62.0 (31.2)	.421
Sm (μm)	113.36 (80.50)	125.48 (78.12)	.611
Tg (μm)	617.31(61.77)	713.87 (68.82)	<.0001
Th (μm)	646.41 (83.40)	631.09 (38.98)	.431
|Tg-Th|(μm)	50.45 (55.61)	85.74 (54.68)	.038

*Post-PK* Post-penetrating keratoplasty, *post-DALK* Post-deep anterior lamellar keratoplasty, *SD* Standard deviation, *IOP* Intraocular pressure, *logMAR BCVA* Logarithm of minimum angle of resolution best-corrected visual acuity, *SE* Spherical equivalent diopter, *DS* Diopter of spherical power, *DC* Diopter of cylindrical power, *GHT* Mean graft-host touch, *Pm* Total prevalence of malapposition proportion, *Sm* Size of malapposition, *Tg* Junctional graft thickness, *Th* Junctional host thickness

**Table 3:** Comparisons of frequency of appositions (F) between Post-PK and Post-DALK groups

Types of apposition	F (%) in Post-PK	F (%) in Post-DALK	P
	Mean (SD), number	Mean (SD), number	
Regular-apposition	45.45(30.50), 80	38.04(31.19), 70	0.425
Step	26.14(21.10), 47	59.28(31.81), 111	<.0001
Graft step	11.36(14.39), 20	54.39(34.26), 102	<.0001
Host step	15.34(21.80),27	3.80(9.56), 9	0.018
Protrusion	23.30(21.58), 41	0.54(2.61),1	<.0001
Hill	17.05(18.32), 33	0(0),0	<.0001
Tag	4.55(7.26), 8	0.54(2.61), 1	0.017
Gape	4.55(7.26), 8	1.09(3.60), 2	0.052

*Post-PK* Post penetrating keratoplasy, *post-DALK* Post-deep anterior lamellar keratoplasty, *F*c Frequency of apposition

Combined data from both post-PK and post-DALK cohorts were used for the following analysis. Although GHIs had quite smooth epithelial surfaces in all scans, malappositions were found on the internal surfaces in 58.3% of scans. In the complete set of internal GHIs, there were 43.9% steps (122 graft steps and 36 host steps), 11.7% protrusions (33 hills and 9 tags), and 2.8% gapes (10 scans). GHT values varied with alignment types as follows: compared with the regular-apposition, internal step and gape significantly reduced GHT, whereas the opposite effect was observed with the hill and tag patterns (Table 4).

**Table 4:** Prevalence and Size of Various Types of Apposition of the Internal Graft-host Interface

Types of apposition	Number of junctions	F (%)	GHT mean (SD), range (μm)	Sm mean (SD), range (μm)	GHT comparison P
Regular-apposition	150	41.7	682.42 (133.89), 470~1450	–	–
Step	158	43.9			
Graft step	122	33.9	658.28 (137.62), 480~1870	198.68 (77.58), 80~680	.074
Host step	36	10.0	662.50 (131.40), 490~1040	236.44 (81.44), 110~480	<.0001
Protrusion	42	11.7			
Hill	33	9.2	819.09 (120.73), 530~1190	184.24 (50.87), 100~310	<.0001
Tag	9	2.5	696.67 (155.72), 510~1010	234.44 (70.38), 160~380	<.0001
Gape	10	2.8	600 (167.59), 440~910	285 (188.81), 170~800	<.0001

*F* Frequency of apposition, *GHT* Mean graft-host touch, *SD* Standard deviation, *Sm* Size of malapposition

**Fig. 1:**
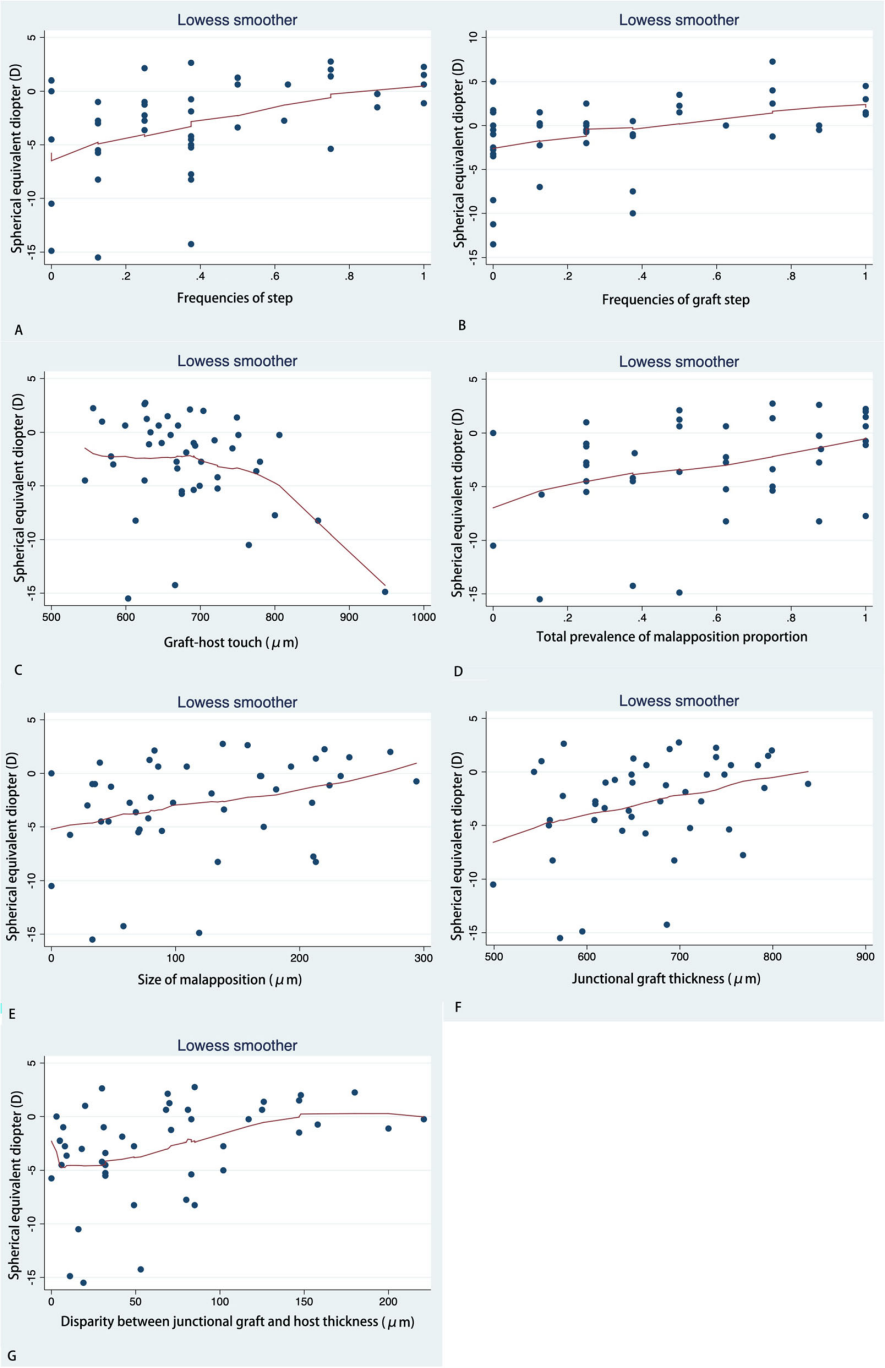
Scatter plots with LOWESS curve. Correlations between spherical equicalent diopter (SE) and frequencies of step (**a**); frequencies of graft step (**b**); graft-host touch (**c**); total prevalence of malapposition proportion (**d**); size of malapposition (**e**); junctional graft thickness (**f**); and Disparity between junctional graft and host thickness (**g**)

**Fig. 2:**
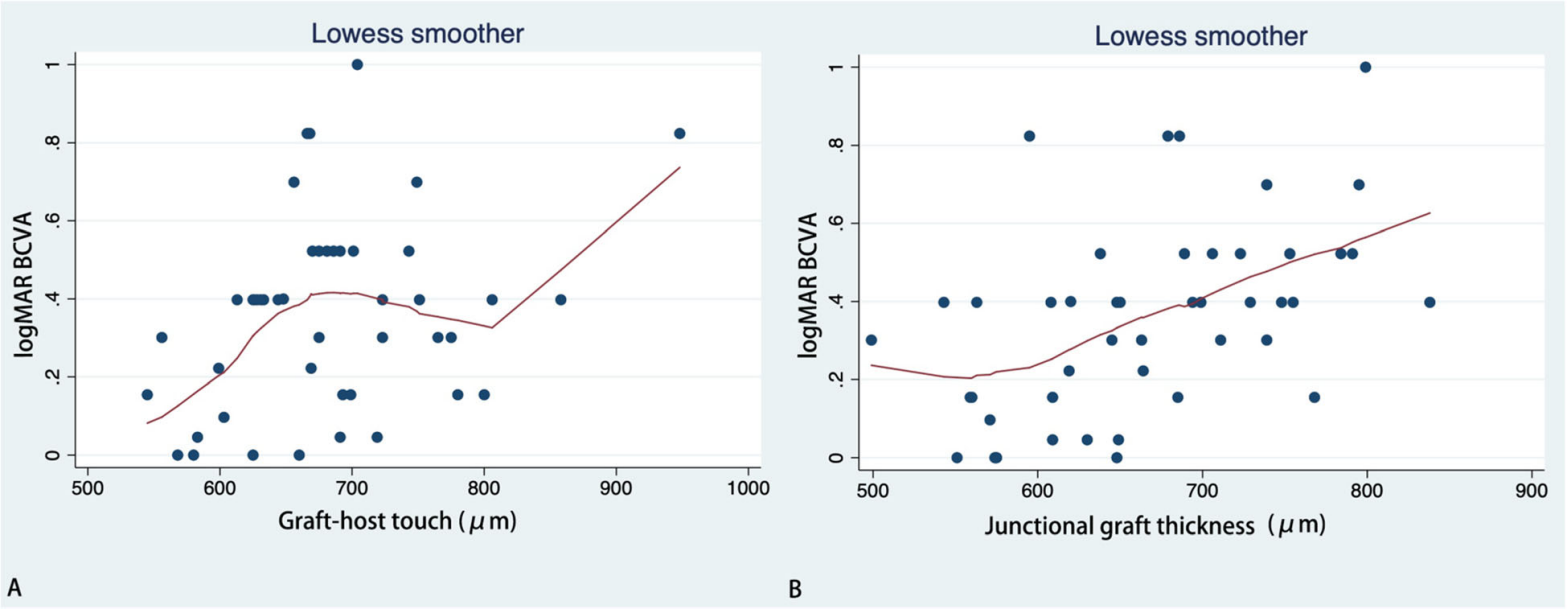
Scatter plots with LOWESS curve. Correlations between the logarithm of minimum angle of resolution best-corrected visual acuity (logMAR BCVA) and graft-host touch (**a**); and junctional graft thickness (**b**)

**Fig. 3:**
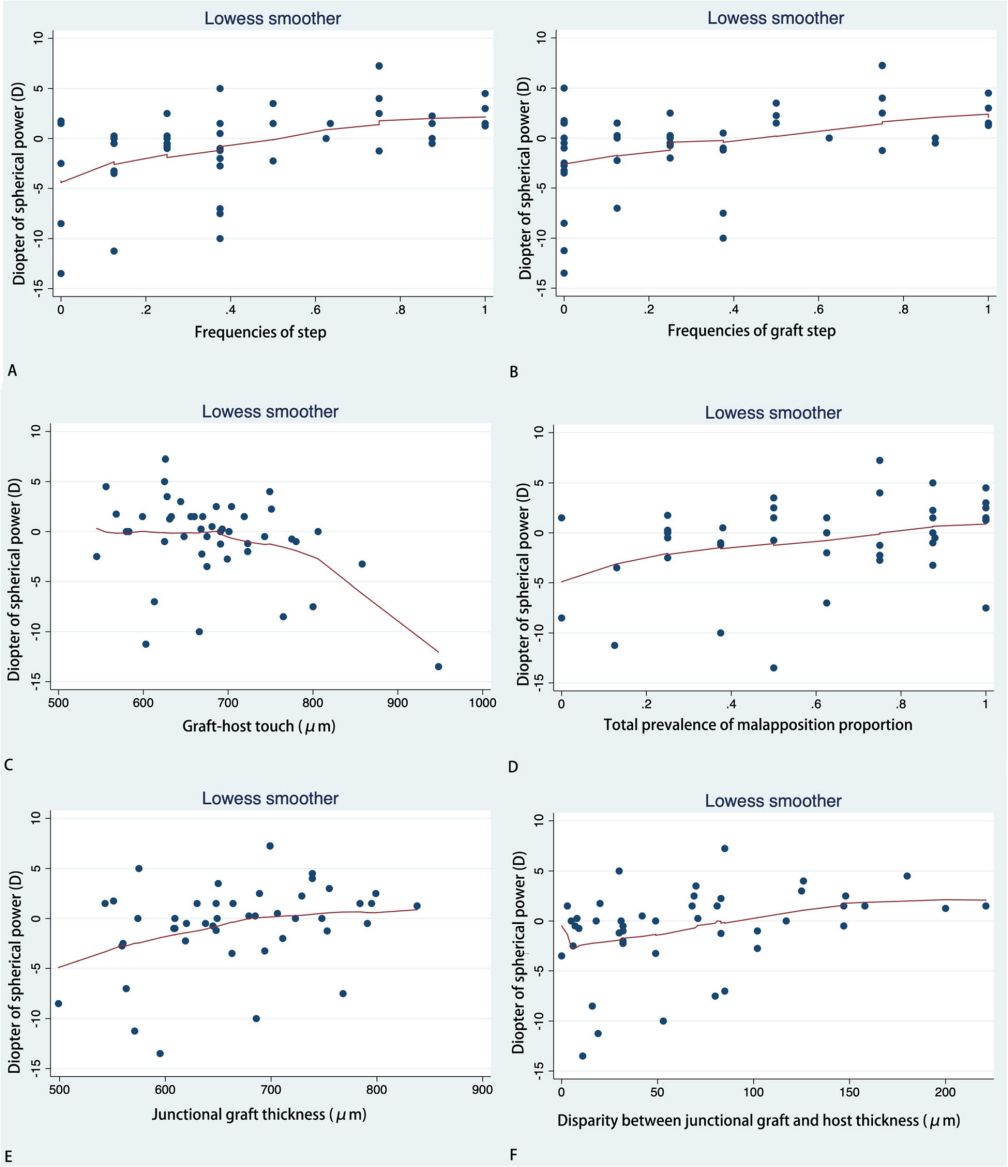
Scatter plots with LOWESS curve. Correlations between diopter of spherical power (DS) and frequencies of step (**a**); frequencies of graft step (**b**); graft-host touch (**c**); total prevalence of malapposition proportion (**d**); junctional graft thickness (**e**); and Disparity between junctional graft and host thickness (**f**)

**Fig. 4:**
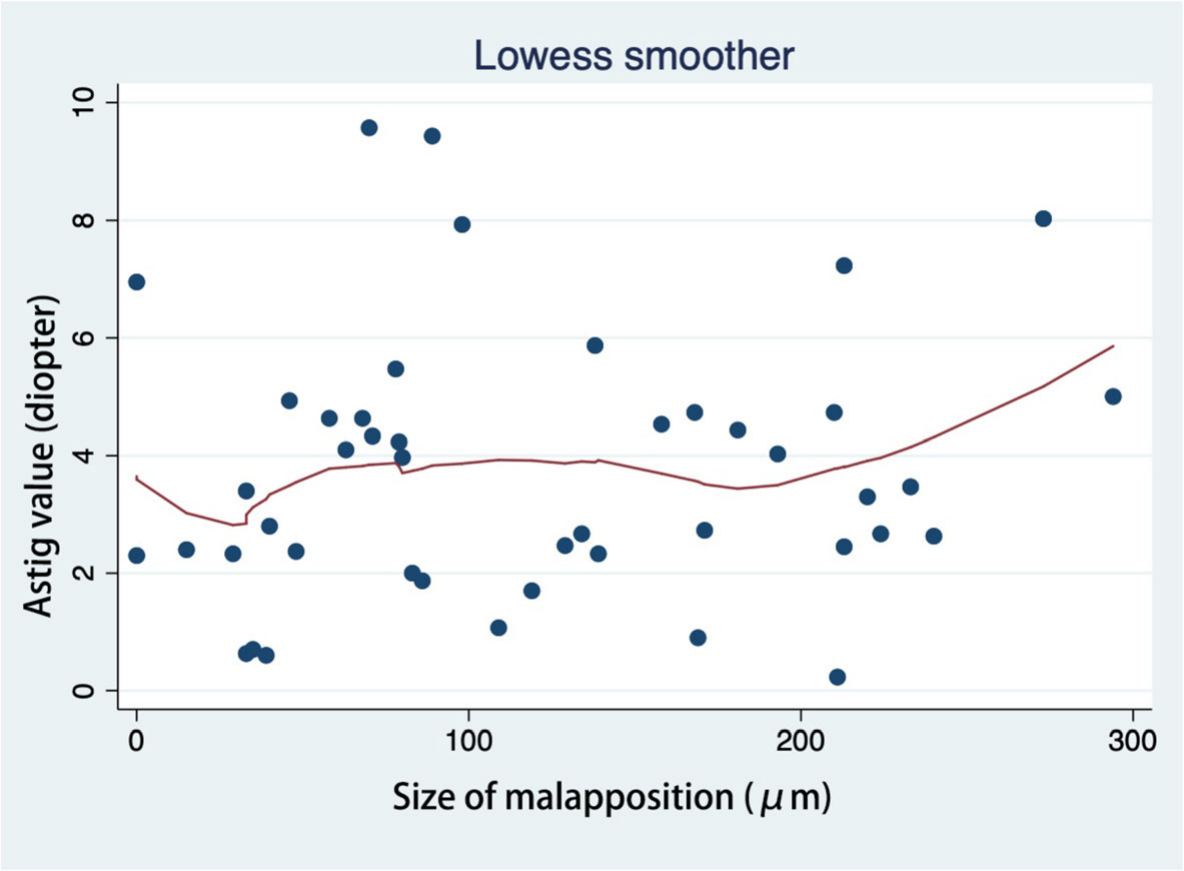
Scatter plots with LOWESS curve. Correlations between the Astig value and size of malapposition

LogMAR BCVA showed a positive correlation with GHT (*r* = 0.030) and Tg (*r* = 0.001), but a negative correlation with the frequency of protrusion [F (protrusion)] (*r* = 0.01). SE was found to have a positive correlation with F (step) (*r* = 0.001), F (graft step) (*r* = 0.028), Pm (*r* = 0.018), Sm (*r* = 0.037), Tg (*r* = 0.022), and |Tg-Th| (*r* = 0.007). SE was negatively correlated with GHT (*r* = 0.021). Very similar results were found for DS. We did not find any relationships between DS and the OCT parameters. Astig value, representing keratometric astigmatism, was found to have slight positive correlation with Sm (*r* = 0.047). Details are listed in Table 5. Scatter plots with LOWESS curves are listed in Fig 1, 2, 3 and 4.

**Table 5:** Correlation Analysis between Graft-host Characteristics and Visual Outcome Parameters

	SE correlation index (*P*)	logMAR BCVA correlation index (*P*)	DS correlation index (*P*)	DC correlation index (*P*)	Astig value correlation index (*P*)
F (step)	0.46 (0.001)	0.24 (0.113)	0.46 (0.002)	0.12 (0.427)	0.22 (0.142)
F (graft step)	0.40 (0.028)	0.33 (0.075)	0.41 (0.024)	0.03 (0.859)	−0.01 (0.973)
F (host step)	0.37 (0.160)	− 0.16 (0.565)	0.37 (0.157)	0.15 (0.582)	0.09 (0.739)
F (protrusion)	−0.29 (0.055)	− 0.38 (0.010)	− 0.29 (0.051)	0.04 (0.820)	0.09 (0.578)
F (hill)	−0.36 (0.186)	0.01 (0.969)	−0.27 (0.332)	−0.32 (0.242)	− 0.22 (0.432)
F (tag)	0.58 (0.134)	−0.42 (0.297)	0.58 (0.134)	−0.25 (0.552)	0.41 (0.310)
F (gape)	0.07 (0.860)	−0.14 (0.720)	−0.13 (0.724)	0.14 (0.724)	0.41 (0.272)
GHT	−0.34 (0.021)	0.32 (0.030)	−0.38 (0.010)	0.00 (0.989)	0.02 (0.908)
Pm	0.35 (0.018)	0.08 (0.609)	0.32 (0.027)	0.12 (0.451)	0.13 (0.395)
Sm	0.31 (0.037)	0.14 (0.377)	0.28 (0.061)	0.17 (0.253)	0.30 (0.047)
Tg	0.03 (0.022)	0.49 (0.001)	0.33 (0.025)	0.10 (0.502)	0.09 (0.538)
Th	0.05 (0.741)	−0.04 (0.779)	0.07 (0.647)	−0.05 (0.735)	0.11 (0.483)
|Tg-Th|	0.40 (0.007)	0.10 (0.522)	0.36 (0.015)	0.21 (0.165)	0.25 (0.102)

*SE* Spherical equivalent diopter, *logMAR BCVA* Logarithm of minimum angle of resolution best-corrected visual acuity, *DS* Diopter of spherical power, *DC* Diopter of spherical power, *F* Frequency of apposition, *GHT* Mean graft-host touch, *Pm* Total prevalence of malapposition proportion, *Sm* Size of malapposition, *Tg* Junctional graft thickness, *Th* Junctional host thickness

Based on the graft-host characteristics with significant correlations, linear regression equations for the visual outcomes were successfully established, except for the one between logMAR BCVA and F (protrution) (F = 3.937, *P* = 0.054). SE increased on average by 6.851, 5.428 and 5.164 diopter per 1% increase in F (step), F (graft step) and Pm, respectively. SE also increased on average by 0.31 for every 10-μm increment in |Tg-Th|. LogMAR BCVA increased on average by 0.01 for each 10-μm increment in both GHT and Tg. DS increased on average by 6.319, 5.067 and 4.518 diopter for every 1% increase in F (step), F (graft step) and Pm, respectively. DS decreased on average by 0.2 diopter per 10-μm increment in GHT. With each 10-μm increase in |Tg-Th|, DS increased by 0.27 diopter averagely. Astig value increased averagely by 0.17 diopter for each 10-μm increment in Sm. Complete linear regression equations are listed in Table 6.

**Table 6:** Linear Regression Analysis of Visual Outcomes

Dependent variable (Y)	Independent variable (X)	Coefficient of regression β (*P*)	Constant α (*P*)	Linear regression equation	95% confidence interval (CI) of β
SE (diopter)	F (step)	6.851 (.001)	−5.850 (<.0001)	Y = 6.851X-5.850	2.975–10.727
	F (graft step)	5.428 (0.005)	−4.708 (<.0001)	Y = 5.428X-4.708	1.685–9.171
	GHT	−0.019 (0.021)	10.391 (0.069)	Y = -0.019X + 10.391	−0.036 - -0.003
	Pm	5.164 (0.018)	−5.912 (<.0001)	Y = 5.164X-5.912	0.913–9.416
	Sm	0.018 (0.037)	−5.046 (<.0001)	Y = 0.018X-5.046	0.001–0.035
	Tg	0.019 (0.022)	−15.588 (0.006)	Y = 0.019X-15.588	0.003–0.035
	|Tg-Th|	0.031 (0.007)	−5.501 (<.0001)	Y = 0.031X-5.501	0.009–0.054
logMAR BCVA	F (protrusion)	–	–	–	
	GHT	0.001 (0.030)	−0.312 (0.309)	Y = 0.001X-0.312	0–0.002
	Tg	0.001 (0.001)	−0.615 (0.028)	Y = 0.001X-0.615	0.001–0.002
DS (diopter)	F (step)	6.319 (0.001)	−3.459 (0.001)	Y = 6.319X-3.459	2.698–9.940
	F (graft step)	5.067 (0.005)	−2.427 (0.005)	Y = 5.067X-2.427	1.582–8.522
	GHT	−0.02 (0.010)	12.948 (0.015)	Y = -0.02X + 12.948	−0.035 - -0.005
	Pm	4.518 (0.027)	−3.374 (0.013)	Y = 4.518X-3.374	0.526–8.511
	Tg	0.017 (0.025)	−12.336 (0.018)	Y = 0.017X-12.336	0.002–0.033
	|Tg-Th|	0.027 (0.015)	−2.564 (0.009)	Y = 0.027X-2.564	0.006–0.048
Astig value (diopter)	Sm	0.017 (0.047)	−1.598 (0.176)	Y = 0.017X-1.598	0–0.033

*CI* Confidence interval, *SE* Spherical equivalent diopter, *DS* Diopter of spherical power, *GHT* Graft-host touch, *logMAR BCVA* Logarithm of minimum angle of resolution best-corrected visual acuity, *Tg* Junctional graft thickness, *F (step)* Frequency of step, *F (graft step)* Frequency of graft step, *Pm* Total prevalence of malapposition proportion, *Sm* Size of malapposition, *|Tg-Th|* Disparity between junctional graft and host thickness, *Sm* Size of malapposition

## Discussion

We found that misalignments of internal GHIs existed in both postsurgical groups, similar to those in previous reports [12, 13]. Smoothness of the anterior GHI can result from several factors. First, the surgeon can align the epithelial surface of a corneal wound under direct vision during an operation. Other factors include suture traction and the powerful regeneration capacity of corneal epithelium. It has been reported that posterior GHI discontinuity exists universally, but related studies are very limited [13]. Lang et al. [15] found misalignments of the DM layer in 22 eyes in post-mortem examinations of 25 patients (30 eyes) who had undergone PK. Kaiserman et al. [12] reported more post-PK internal graft-host malappositions (76.4%) in keratoconic patients compared with those who received corneal transplantations for other corneal diseases (50%). For keratoconic patients, the higher probability of irregular wound healing might be due to uneven thinning of the cornea before surgery. In our study, 58.3% graft-host junctions were misaligned. The major reasons for the relatively low incidence of misalignment may be the expertise of our experienced surgeons and the use of equal graft-host size. Moreover, unlike previous studies, we enrolled both post-PK and post-DALK patients. The different healing processes of these two procedures might account for the discrepancy in misalignment rates among studies [16].

Until now, there have been conflicting opinions about the causes of postoperative astigmatism; these include small intraoperative trephinations, uneven suture tension and graft-recipient misalignments [8, 11]. Limberg et al. [17] proposed that imprecise graft-host matching might result in astigmatism of about 4 to 6 diopters [1]. Kaiserman et al. [11] reported slight misalignment-associated astigmatism after PK. Jhanji et al. [18] categorized the alignment patterns as step and ledge, but failed to describe the relevance of malappositions to postoperative visual outcomes. However, they proposed a theory that an oversized graft would affect the GHI alignment due to the curled shape of the internal surface of the larger graft. In the current study, graft and host beds were prepared isometrically in all cases so size difference was not an issue. Suture tension within each case proved to be almost even, based on the symmetry of postoperative corneal morphology under AS-OCT observation.

Referring to lamellar keratoplasty, the GHI manifest as a moderate-to- highly reflective interface in AS-OCT images [19]. However, the incision depth of corneal stroma in our DALK procedure virtually reached the DM layer. Hence, the very thin residual stroma made the AS-OCT cross-sectional images from the PK and DALK groups very similar [19]. In our study, the PK group had better logMAR BCVA than did DALK group [(0.25 ± 0.24) vs. (0.47 ± 0.20), *P* = 0.002] despite similar astigmatism. This is probably due to the shorter postoperative recovery time in the DALK cohort, resulting in thicker junctional graft, with the consequent irregular or optically less clear GHI resulting in lower visual acuity. There have been different opinions about the relative merits of these two surgeries for years. However, there was strong evidence, from a register of controlled trials, suggesting better logMAR BCVA at ≥6 months with PK in a recent systematic review [4]. Our results concur with that conclusion [10]. The more severe SE and DS results in our study might be attirubuted to the relatively worse keratoconus and higher degree of myopia before surgery in our patients [2].

Post-keratoplasty alignment patterns can be classified into four basic types: regular apposition, step, protrusion and gape. Moreover, graft step and host step are subtypes of step, while hill and tag are subtypes of protrusion [12]. Studies have shown that various preoperative corneal pathologies influence the wound alignment patterns [12, 20]. Sung et al. [13] used AS-OCT to observe the posterior surface of corneal wounds from 13 post-PK keratoconic eyes, and found 78.8% had malapposed junctions, including 22.1% with gape, 22.1% protrusion and 34.6% step. Among the 360 graft-host sections, confined to only keratoconic eyes in the current study, the most common malapposition was graft step (122 cases, 33.9%). Since it has been reported that preoperative corneal pathology can influence the graft-host apposition patterns [14, 21], we hold the opinion that asymmetrical pre-operative thinning of the cornea in different disease stages caused increased graft step numbers, because the normal corneal grafts from donors were generally thicker than the recipient beds, which had already thinned.

GHT represented the contact area between the graft and recipient bed. Generally, step and gape significantly reduced GHT, whereas hill increased GHT. In the current study, tag pattern also slightly increased GHT. “Tag” refers to a small piece of DM layer protruding from the corneal wound, while “hill” is a protrusion of both the DM layer and deep corneal stroma [12, 13]. Hence, we think that tag should increase GHT, but only to a very limited extent.

In present study, various associations between visual outcomes and characteristics of the corneal alignment patterns were specifically evaluated and quantified. Generally, we found the malappositions that decreased GHT significantly increased the postoperative SE and DS. However, the decreased GHT lowered logMAR BCVA. We speculate that expansion of GHT might enhance stability of the corneal wound, and thus reduce the postoperative SE and DS. However, logMAR BCVA increased slightly with GHT in some unknown way, which needs further exploration. SE and DS significantly increased under the influence of F (step), F (graft step), Pm, Tg, and |Tg-Th|. Specifically, Astig value and SE both showed a slight tendency to increase along with increments of Sm. We speculate that F (step), F (graft step) and Pm may be the major factors responsible for post-keratoplasty ametropia. Moreover, the relationships between |Tg-Th| and visual outcomes indicated that larger graft-host disparity might lead to more-serious ametropia. Hence, we assume that the thinner the recipient bed is, the worse the visual outcomes will be. The increase in graft thickness mainly occurs shortly after surgery, because of tissue edema; hence, this could explain the increase of logMAR BCVA, SE, and DS with Tg, because most cases were observed shortly after operation. Sung et al. [13] evaluated the characteristics of GHI after PK using AS-OCT and reported that the graft-host thickness disparity, which closely related to the wound alignment state, showed a positive correlation with keratometric astigmatism (*r* = 0.56, *P* < 0.01). Kaiserman et al. [12] analyzed 204 post-PK graft-host sections from 27 eyes, and found that Sm correlated negatively with postoperative SE (*r* = − 0.2, *P* = 0.02) and positively with postoperative DC (*r* = 0.26, *P* = 0.006). Although the correlations were slight, Sm could explain, in part, the astigmatism and ametropia. Herein, Sm increase the degree of astigmatism, if only to a minor extent.

Our study has several limitations. Because it only represented a single-center experience, with relatively few subjects, we may have missed some statistical conclusions that might otherwise have been significant. Next, the study subjects were restricted only to keratoconic patients, so it may not be possible to extrapolate from our results to cases with other corneal diseases. Moreover, we unavoidably missed some information about internal GHIs because data for only eight positions per eye were acquired within a raster scan.

We verified the widespread existence of internal graft-host malappositions using AS-OCT and quantified the relationships between GHI characteristics and visual outcomes in postoperative keratoconic patients for the first time. GHT increased in protrusion alignment and decreased in step and gape patterns. F (step), F (graft step), Pm and |Tg-Th| influenced SE and DS positively. Central corneal keratometric astigmatism increased along with Sm increment. LogMAR BCVA rose with increasing GHT and Tg.

## Conclusions

In conclusion, investigating of the characteristics of GHI is valuable for explaining varied ametropia in keratoconic eyes. Our study has potential reference value for future technological advancement. Further studies are warranted to determine better ways of achieving optimal graft-host apposition.

## Supplementary information

**Additional file 1: Figure S1**. Schematic diagram depicting eight corneal graft-host interface (GHI) (aqua spots) points in frontal view.

**Additional file 2: Figure S2**. Graft-host alignment patterns observed using AS-OCT, with schematic diagrams. (A) regular-apposed junction; (B) graft-step pattern; (C) host-step pattern; (D) hill pattern; (E) tag pattern; (F) gape pattern. The red solid and dotted lines represent the graft-host interface. The grey curves represent Descemet’s layer.

**Additional file 3: Figure S3**. Measurement method for graft and host thickness (Tg and Th) at the corneal wound interface (A). Assuming that the red spots and yellow spots are two different types of malapposition, Pm in this case would be [(2 + 2)/8]*100%, and F (red spot malapposition) would be (2/8)*100% (B).

**Additional file 4: Figure S4**. Schematic diagrams depicting measurement methods for GHT and Sm in six types of apposition. The aqua spots represent external and internal junction points; red solid and dotted lines represent GHT; blue lines represent Sm. (A) regular-apposed junction; (B) graft-step pattern; (C) host-step pattern; (D) hill pattern; (E) tag pattern; (F) gape pattern.

**Additional file 5: Figure S5**. Bar-chart comparing frequencies of varied appositions between the post-PK and post DALK group.

## Data Availability

All data generated or analyzed during this study are included in this published article.

## Abbreviations

|Tg-Th|: Disparity between junctional graft and host thickness
AS-OCT: Anterior-segment optical coherence tomography
CI: Confidence interval
DALK: Deep anterior lamellar keratoplasty
DC: Diopter of cylindrical power
DM: Descemet’s membrane
DS: Diopter of spherical power
F (graft step): Frequency of graft step
F (step): Frequency of step
F: Frequency of apposition
GHI: Graft-host interfaces
GHT: Graft-host touch
IOP: Intraocular pressure
logMAR BCVA: Logarithm of minimum angle of resolution best-corrected visual acuity
PK: Penetrating keratoplasty
Pm: Total prevalence of malapposition proportion
post-DALK: Post-deep anterior lamellar keratoplasty
post-PK: Post- penetrating keratoplasty
SD: Standard deviation
SE: Spherical equivalent diopter
Sm: Size of malapposition
Tg: Junctional graft thickness
Th: Junctional host thickness
