# Supplementary figures and images for: Malapposition of graft-host interface after penetrating keratoplasty (PK) and deep anterior lamellar keratoplasty (DALK): an optical coherence tomography study
Source: BMC Ophthalmol. 2020 Jan 31;20:41. doi: 10.1186/s12886-020-1307-7 (PMC6995222; doi:10.1186/s12886-020-1307-7)

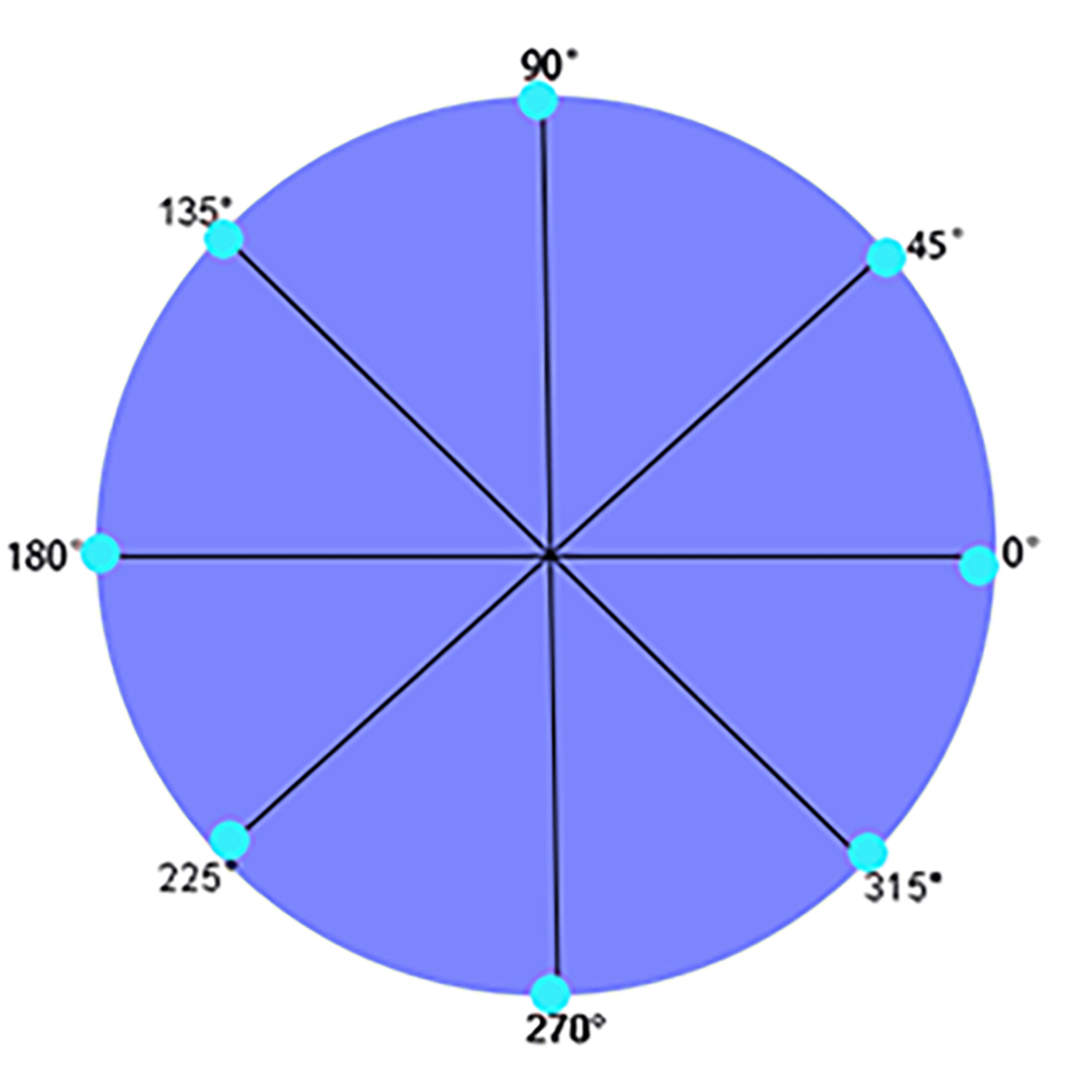

Supplement: Supplementary file 1 — Additional file 1: Figure S1. Schematic diagram depicting eight corneal graft-host interface (GHI) (aqua spots) points in frontal view. [file 12886_2020_1307_MOESM1_ESM.tif]

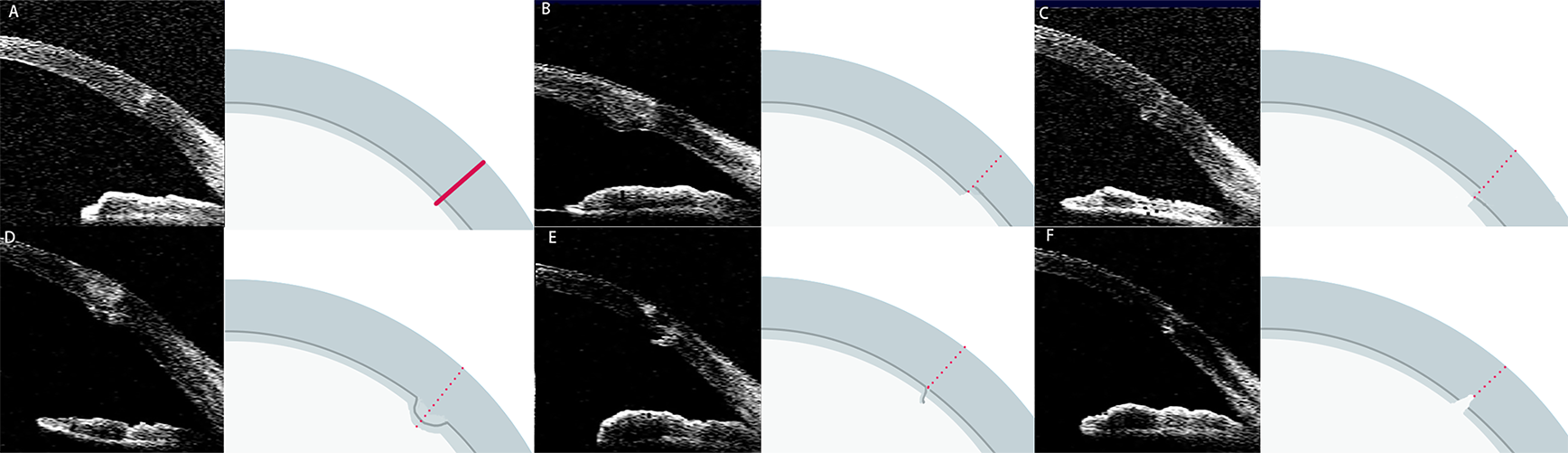

Supplement: Supplementary file 2 — Additional file 2: Figure S2. Graft-host alignment patterns observed using AS-OCT, with schematic diagrams. (A) regular-apposed junction; (B) graft-step pattern; (C) host-step pattern; (D) hill pattern; (E) tag pattern; (F) gape pattern. The red solid and dotted lines represent the graft-host interface. The grey curves represent Descemet’s layer. [file 12886_2020_1307_MOESM2_ESM.tif]

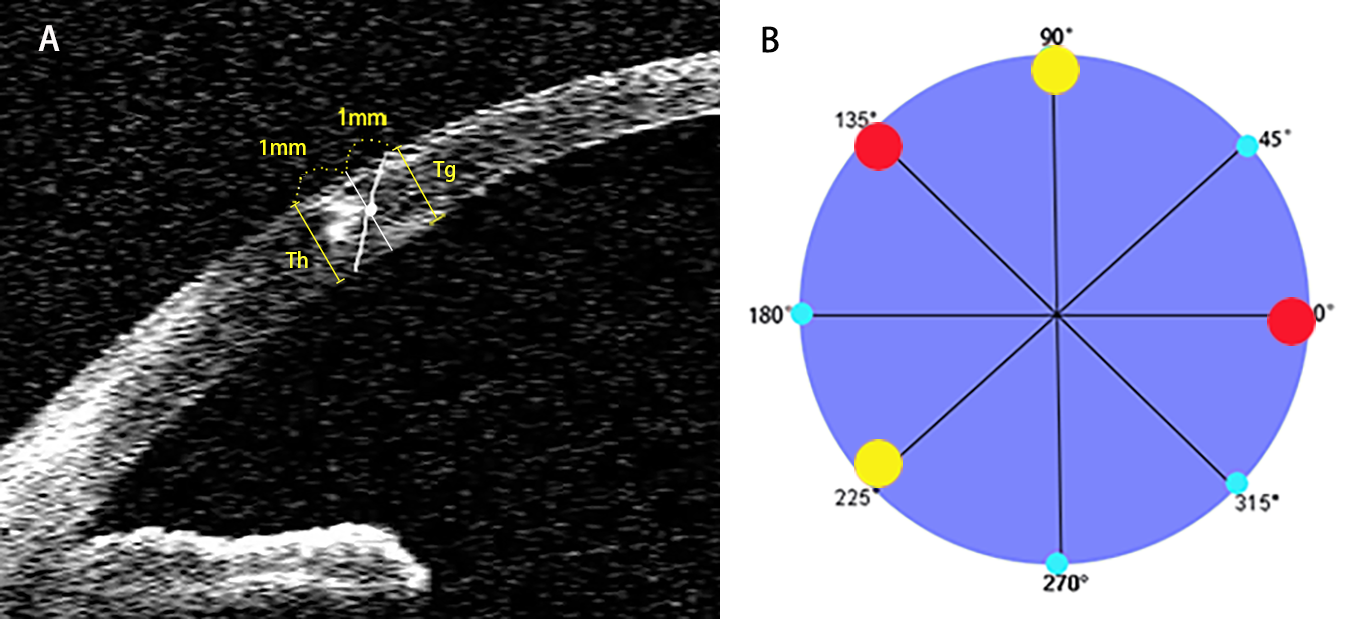

Supplement: Supplementary file 3 — Additional file 3: Figure S3. Measurement method for graft and host thickness (Tg and Th) at the corneal wound interface (A). Assuming that the red spots and yellow spots are two different types of malapposition, Pm in this case would be [(2 + 2)/8]*100%, and F (red spot malapposition) would be (2/8)*100% (B). [file 12886_2020_1307_MOESM3_ESM.tif]

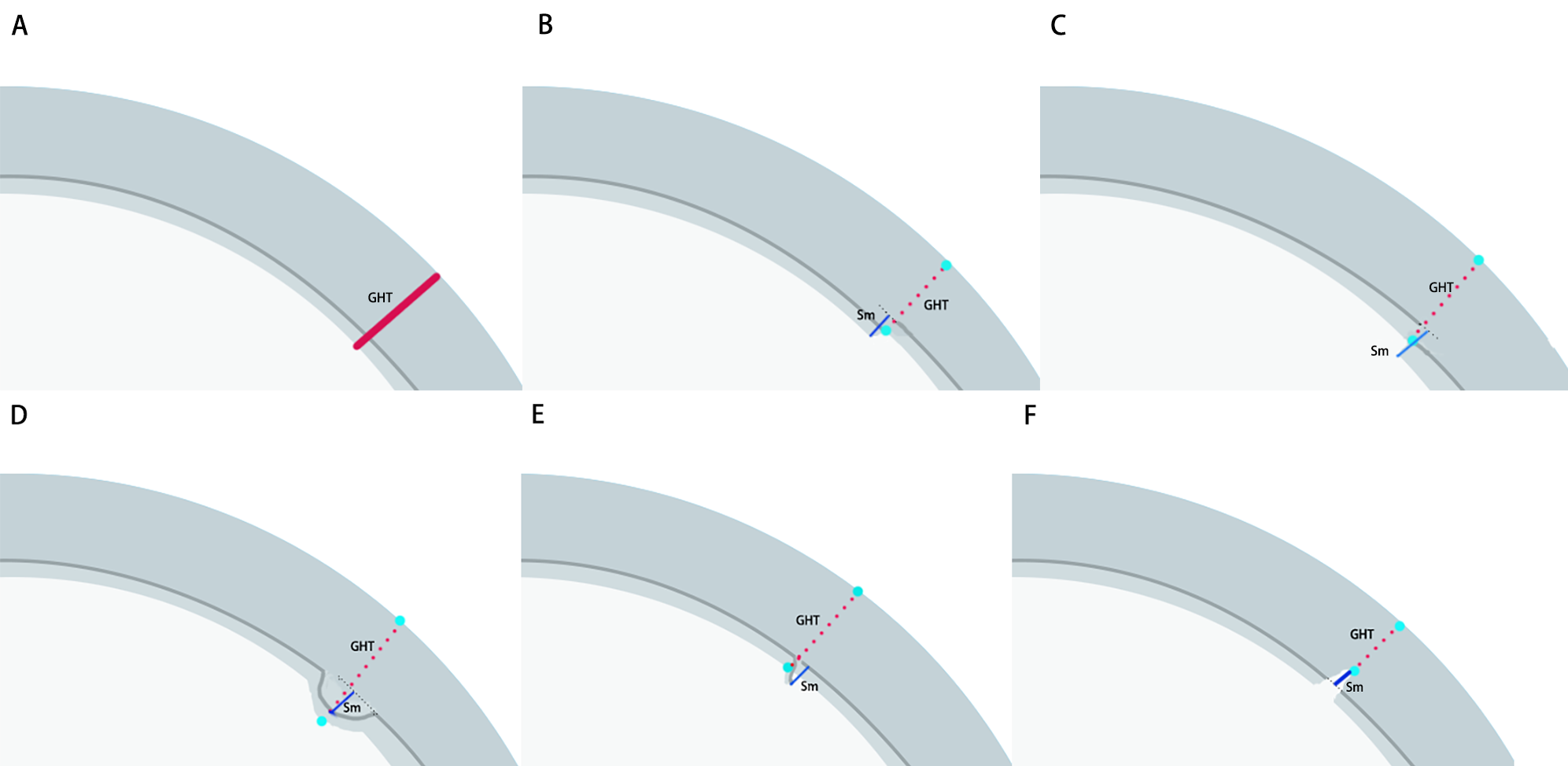

Supplement: Supplementary file 4 — Additional file 4: Figure S4. Schematic diagrams depicting measurement methods for GHT and Sm in six types of apposition. The aqua spots represent external and internal junction points; red solid and dotted lines represent GHT; blue lines represent Sm. (A) regular-apposed junction; (B) graft-step pattern; (C) host-step pattern; (D) hill pattern; (E) tag pattern; (F) gape pattern. [file 12886_2020_1307_MOESM4_ESM.tif]

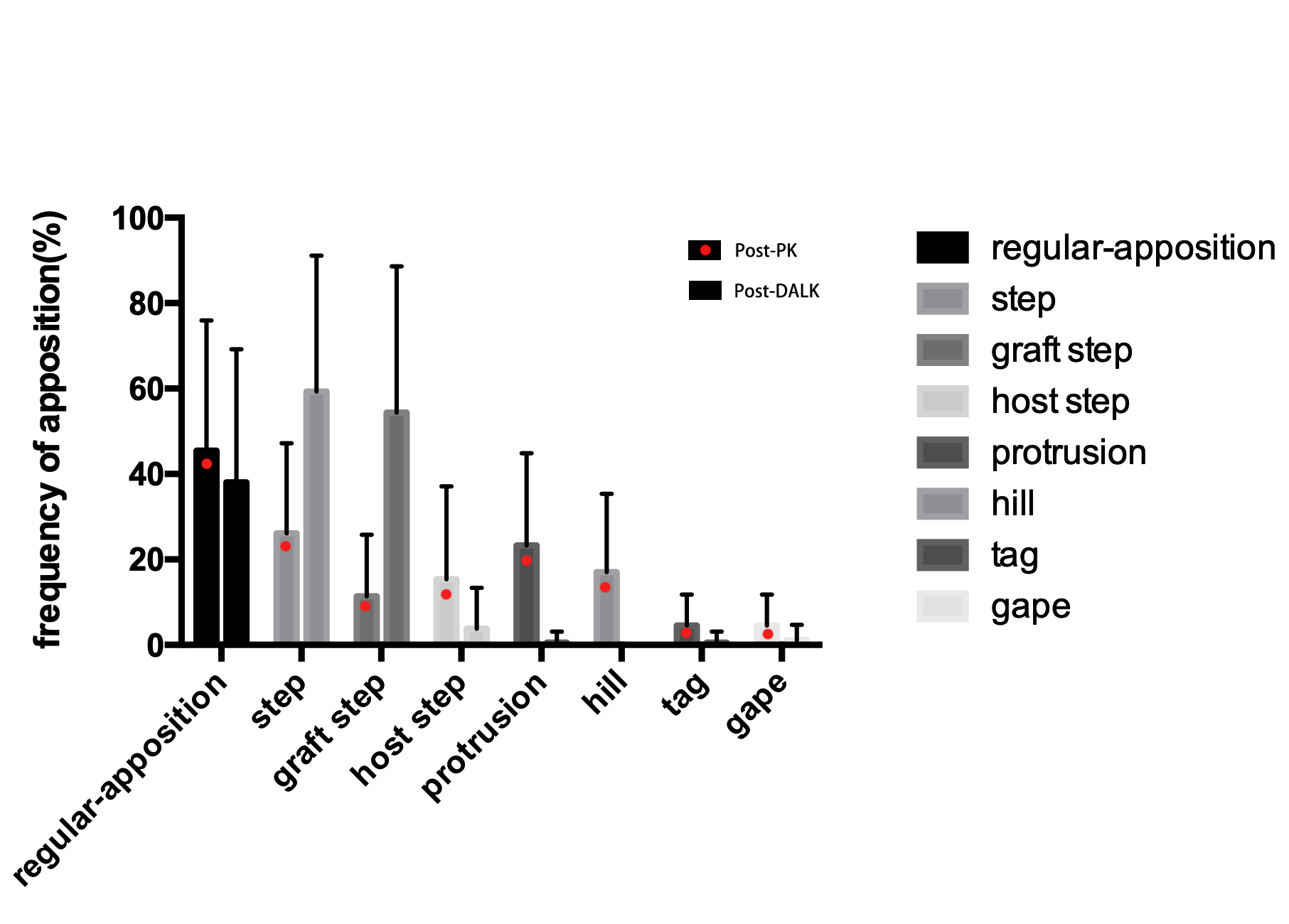

Supplement: Supplementary file 5 — Additional file 5: Figure S5. Bar-chart comparing frequencies of varied appositions between the post-PK and post DALK group. [file 12886_2020_1307_MOESM5_ESM.tif]
